# Tumor and Immune Dynamics Following Sequential CDK4/6 and PD-1 Inhibition: Results from a Phase 2 Study in Dedifferentiated Liposarcoma

**DOI:** 10.1158/2767-9764.CRC-25-0334

**Published:** 2026-02-27

**Authors:** Evan Rosenbaum, Rodrigo Gularte-Mérida, Evan Seffar, Jasme Lee, Mathew Adamow, Martina Bradic, Mark A. Dickson, Viswatej Avutu, Lauren B. Banks, Jason E. Chan, Ping Chi, Mrinal M. Gounder, Ciara M. Kelly, Mary Louise Keohan, Robert G. Maki, Sujana Movva, Damon R. Reed, Rhoena Desir, Matthew Biniakewitz, Joseph P. Erinjeri, Robert A. Lefkowitz, Phillip Wong, Cristina R. Antonescu, Li-Xuan Qin, Katherine S. Panageas, Ronglai Shen, Samuel Singer, Andrew Koff, William D. Tap, Sandra P. D’Angelo

**Affiliations:** 1 Department of Medicine, Memorial Sloan Kettering Cancer Center, New York, New York.; 2 Department of Medicine, Weill Cornell Medical College, New York, New York.; 3 Department of Surgery, Memorial Sloan Kettering Cancer Center, New York, New York.; 4 Department of Epidemiology and Biostatistics, Memorial Sloan Kettering Cancer Center, New York, New York.; 5 Immune Monitoring Facility, Memorial Sloan Kettering Cancer Center, New York, New York.; 6 Center for Molecular Oncology, Memorial Sloan Kettering Cancer Center, New York, New York.; 7 Department of Pediatrics, Memorial Sloan Kettering Cancer Center, New York, New York.; 8 Department of Radiology, Memorial Sloan Kettering Cancer Center, New York, New York.; 9 Department of Pathology, Memorial Sloan Kettering Cancer Center, New York, New York.; 10 Sloan Kettering Institute, Memorial Sloan Kettering Cancer Center, New York, New York.

## Abstract

**Purpose:**

The CDK4/6 inhibitor palbociclib delays progression in patients with advanced dedifferentiated liposarcoma (DDLPS). In carcinoma models, CDK4/6 and PD-1 inhibition induce intratumoral inflammation and synergistic activity. Comprehensive assessment of tumor and host dynamics is needed to understand response and resistance to this combination.

**Patients and Methods:**

We performed a phase 2 study of palbociclib and the PD-1 inhibitor retifanlimab in advanced DDLPS. Palbociclib was administered 2 weeks prior to retifanlimab. Tumor biopsies were analyzed by single-cell RNA sequencing (scRNA-seq), and peripheral blood by high-parameter flow cytometry. Analyses focused on cell cycle, senescence, and immune-related changes.

**Results:**

Twelve patients were treated before the study was halted because of a 42% immune-related toxicity rate. The overall response rate was 8.3%, and the disease control rate was 75%. Median progression-free and overall survival rates were 7.1 and 26.8 months, respectively. Nine patients had at least one tumor biopsy analyzed; three had paired. Ten patients had paired blood samples analyzed. Following treatment, tumor cells demonstrated decreased cycling and increased cell-cycle checkpoint activity. Transcriptional scores for senescence increased in cancer cells, as did the proportion of intratumoral T and B cells. A cluster of cells emerged with upregulated cell-cycle genes and downregulated HLA class I, suggesting innate or acquired resistance to treatment. In blood, a subset of CD4^+^ T cells decreased, whereas expression of LAG-3, ICOS, and CD38 increased in select subsets.

**Conclusions:**

A palbociclib lead-in prior to retifanlimab had a high rate of immune-related toxicities. Correlative analyses identified changes in tumor and immune cells attributable to treatment. A study of concurrent dosing of the combination is ongoing.

**Significance:**

In this phase 2 study of palbociclib and retifanlimab, treatment led to immune-related adverse events, halting enrollment. scRNA-seq and flow cytometry revealed cell-cycle suppression, senescence, and T-cell changes. These findings highlight the rationale for this combination and the need for safer dosing strategies in larger cohorts.

## Introduction

Dedifferentiated liposarcoma (DDLPS) is an aggressive histologic subtype of soft tissue sarcoma that frequently arises in the retroperitoneum or extremities (1). Surgical resection is the treatment of choice, yet local recurrences are common and often become unresectable, turning deadly for patients (2, 3). Systemic therapies have only modest efficacy, with anthracyclines achieving an overall response rate of <20% of patients (4). The median overall survival (OS) for patients with unresectable or metastatic DDLPS is approximately 2 years (5).

DDLPS is characterized by near-ubiquitous amplification of chromosome 12q13-15, including the oncogenes *CDK4* and *MDM2* (6). Clinical trials of the CDK4/6 inhibitors (CDK4/6i) palbociclib and abemaciclib demonstrated promising efficacy in patients with DDLPS, with progression-free survival (PFS) rates at 12 weeks of 57% and 76%, respectively. Despite these encouraging findings, overall response rates to these agents are low (≤10%; refs. 7–9).

CDK4/6is can induce cellular senescence, a durable cell-cycle exit in proliferating cancer cells, resulting in the release of immunogenic chemokines in an otherwise immune-cold microenvironment (10). Patients with DDLPS treated with palbociclib who achieve therapy-induced senescence show better clinical outcomes (11, 12). Abemaciclib-treated patients with DDLPS who benefit from therapy have elevated expression of a senescence-associated secretory phenotype (SASP) gene signature, including *ANGPTL4* and *CDKN2A*. This signature correlates with increased CD4^+^ tumor-infiltrating lymphocytes, suggesting a connection between SASP and tumor inflammation during abemaciclib therapy (13).

Preclinical studies demonstrate that CDK4/6is can also directly activate antitumor immunity without senescence (11, 14, 15). When combined with immune checkpoint blockade (ICB) in murine models, CDK4/6i therapy leads to tumor regression, increased antigen presentation, and an inflamed T-cell phenotype (16). CDK4/6is can also overcome ICB resistance by reversing innate immune evasion and T-cell exclusion, suggesting their potential to improve ICB efficacy in tumors with low T-cell infiltration (17).

Herein, we describe the clinical and correlative results from a phase 2 study with safety lead-in utilizing palbociclib alone for 2 weeks prior to adding the PD-1 inhibitor retifanlimab in patients with DDLPS. Our analyses provide a framework for using single-cell RNA sequencing (scRNA-seq) combined with high-parameter flow cytometry to generate mechanistic insights into how senescence might participate in the immune response to CDK4/6 plus PD-1 inhibition. We show that there is considerable interpatient variability observed in the DDLPS tumor microenvironment and host adaptive immune responses, highlighting the importance of analyzing senescence in individual tumors sampled before and during treatment.

## Patients and Methods

### Study design and statistical plan

This was a phase II, single-center, investigator-initiated study of palbociclib plus retifanlimab in patients with advanced DDLPS. The study incorporated a safety lead-in of six patients to determine the recommended phase 2 dose (RP2D). Dose-limiting toxicities (DLT) were assessed within the first 6 weeks of treatment. If ≤1 patient of six had a DLT, the dosing schedule used in the lead-in phase would be declared the RP2D. The study was planned to expand and enroll an additional 24 patients, for a total of 30, between both study phases. Key eligibility criteria included having a diagnosis of advanced or metastatic DDLPS, age ≥18 years, Eastern Cooperative Oncology Group performance status of ≤1, measurable by RECIST 1.1, and adequate organ and marrow function. Key exclusion criteria included prior treatment with ICB or CDK4/6 inhibition, symptomatic autoimmune disease within 2 years of enrollment, or active corticosteroid use or other immunosuppressive medications.

**Figure 1.**
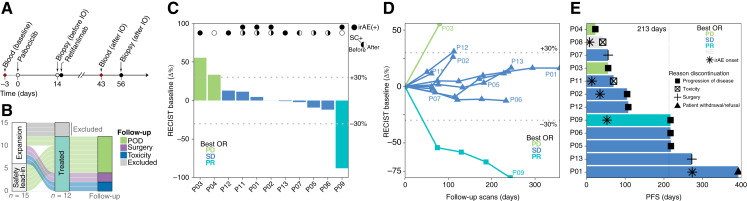
Clinical trial design and efficacy. **A,** Study schema depicting treatment and biopsy collection time points. **B,** Flow diagram of treatment and follow-up for patients in the safety lead-in and trial expansion cohorts. **C,** Waterfall plot showing best percent tumor volume change from baseline per RECIST 1.1. One patient who was not evaluable for response by RECIST 1.1 is excluded. irAE(+), solid circles indicate irAEs; SC+, single-cell data from tumor biopsies, in which filling of the left and right halves indicates analyzability at the pre- and post-retifanlimab time points, respectively. **D,** Spider plot showing percent change in sum of target lesion diameters over time in patients who were evaluable for response by RECIST v1.1. **E,** Swimmer plot showing patient PFS. Dashed line indicates median PFS. NE, not evaluable; OR, overall response.

Palbociclib was administered at the FDA-approved dose of 125 mg orally daily for 21 consecutive days, followed by 7 days off. Palbociclib treatment was initiated on day 1 of each 28-day cycle. Retifanlimab was given on day 15 of each cycle at an intravenous flat dose of 500 mg (Fig. 1A). Tumor biopsies were obtained on days 14 and 56 of palbociclib treatment, before and after retifanlimab initiation, respectively. Peripheral blood was collected at baseline (before palbociclib) and on-treatment (after ICB initiation), independently of biopsies. The study was conducted in accordance with FDA regulations and Good Clinical Practice guidelines. The study protocol was approved by the Institutional Review Board at Memorial Sloan Kettering Cancer Center (MSKCC), and written informed consent was obtained from all patients participating.

The primary endpoint was to determine the objective response rate (ORR) by RECIST 1.1. Secondary endpoints included describing the safety of the treatment combination and estimating the PFS, OS, and disease control rate. An ORR of 5% ORR was considered not promising and 25% was considered promising. The null hypothesis would be rejected if four or more patients have a confirmed objective response to treatment. This design has a type I error rate of 0.06 and a type II error rate of 0.04. The ORR was determined as the proportion of evaluable patients who have complete response or partial response (PR) defined by RECIST. Safety was assessed using Common Terminology Criteria for Adverse Events v5.0. OS and PFS intervals were estimated using Kaplan–Meier methodology. The duration of response was estimated among responders from the time of response until disease progression by RECIST, using Kaplan–Meier methodology. Patients who did not experience progression or death by the end of the study were censored at the time of the last follow-up.

### scRNA-seq and data analysis

Fresh biopsies were dissociated using 5 mg/mL collagenase IV in Hank’s Balanced Salt Solution (HBSS; Millipore Sigma, cat. #C5138) at 37°C and 220 RPM for 15 to 40 minutes. Incubation was stopped when tissue was fully dissociated. Collagenase was quenched with 20 mL of ice-cold HBSS, and dissociated tissue was passed through a 40-μm filter (Fischer Scientific, cat. #22-363-547) and centrifuged at 450 × *g* for 10 minutes. The supernatant was removed, and the cell pellet was resuspended in 5 mL of PBS with 1% BSA (Ambion, cat. #AM2616) and passed through a 40-μm cell strainer into a polystyrene tube (Falcon, cat. #352235) and centrifuged at 450 × *g* for 5 minutes. Final resuspension was into 500 μL to assess viability and cell concentration via trypan blue. Cell concentration was adjusted to 1,100 cells/μL for 10× sequencing. Samples were then processed following 10X Genomics guidelines for gel bead-in-emulsion library preparation and sequenced using a 10X Genomics Xenium instrument (RRID: SCR_025847) to 25,000 reads on average. Two capture wells per biopsy were prepared to maximize the number of cells.

Resulting single-cell data were using Cell Ranger v7.1 (RRID: SCR_023221) aligned to the GRCh38.p5 reference genome to generate a cell count matrix. Data were filtered to remove putative doublet cells (18) and high mitochondrial expression rates (19). Cell types were classified via Azimuth (RRID: SCR_021084) using the Adipose and peripheral blood mononuclear cell (PBMC) references (20, 21). Cells having <256 or >5,792 expressed genes, with <362 total reads >50% ribosomal expression, >3% hemoglobin genes, >0.7% platelet markers, or nonconcordant predicted cell type between the Adipose and PBMC references were excluded from further analysis. Final data analysis was carried out in Seurat v4 (RRID: SCR_007322) using sctransform (RRID: SCR_022146) with 20,000 cells for subsampling, regressing on the percent mitochondrial expression, and using 35 principal components (22, 23). In addition, cancer cells were classified and quantified using the six liposarcoma cancer cell signatures corresponding to adipocyte differentiation, stemness, extracellular matrix (ECM) remodeling, hypoxia, angiogenesis, and invasion, using their canonical markers (24).

Longitudinal, clone-specific analysis of SASP and other signatures was carried out using only the cancer cells of patients P03 and P12. For this analysis, cancer cells were reclustered using Harmony (RRID: SCR_022206) to ensure correct clone alignment between pre- and post-retifanlimab biopsies (25). Differential gene expression was analyzed using *FindMarkers*, and genes with a Bonferroni-corrected *P* value ≤ 0.01 were considered significant.

### Flow cytometry

PBMCs were processed and analyzed using a 29-color flow panel on a BD FACSymphony at the Immune Monitoring Facility at MSKCC as previously described (26). Specific antibodies used are listed in Supplementary Table S1. The resulting flow samples were processed in R v4.4.1 via the automated gating pipeline *staRgate*, publicly available on GitHub (https://github.com/leejasme/staRgate; ref. 27). Single-cell flow data were summarized as percentages of cells expressing a given marker, in which marker-positive cells represent unique T-cell subpopulations. We descriptively summarized a subset of 63 T-cell subpopulations representing various differentiation, activation, senescence, and exhaustion states (defined by various immune checkpoint molecules) at baseline and on-treatment, as well as their on-treatment changes. More specifically, the analyzed subset consisted of key parent subpopulations of T cells that included CD3^+^ lymphocytes, CD3^+^CD4^+^CD8^−^ and CD3^+^CD4^−^CD8^+^ T cells, as well as regulatory T cells (Treg) defined by positive expression of FoxP3 on CD4^+^ cells. Subpopulations were further broken down into CD4^+^ and CD8^+^ T-cell differentiation states defined by varying coexpression of CD45RA and CCR7 [naïve (Tn): CD45RA^+^ CCR7^+^, central memory (Tcm): CD45RA^−^ CCR7+, effector memory (Tem): CD45RA^−^CCR7^−^, and terminally differentiated (Temra): CD45RA^+^ CCR7^−^]. On these subsets, we examined further the expression of various immune checkpoint molecules involved in exhaustion (LAG-3, CTLA-4, PD-1, TIM-3, and TIGIT), senescence (CD57), and activation (ICOS, HLA-DR, and CD38), as well as a transcriptional regulator of PD-1 repression (Tbet). Changes were analyzed via Wilcoxon signed-rank test with FDR adjustment for multiple comparisons. The results were visualized using boxplots and bar graphs, with bar graphs scaled by the SDs

### Statistical analysis

All data were analyzed and visualized using R v4.4.1 (RRID: SCR_001905).

## Results

### Safety and efficacy of palbociclib and retifanlimab in patients with DDLPS

Between June 2020 and December 2021, 15 patients were enrolled and 12 were treated with palbociclib plus retifanlimab (Table 1; Supplementary Tables S2 and S3). Three patients were not treated due to ineligibility or consent withdrawal (Fig. 1B). One of six patients treated on the safety lead-in experienced a DLT of grade 3 febrile neutropenia that resolved with supportive care. The study was expanded, and an additional six patients were enrolled and treated. All patients had at least one treatment-related adverse event (TRAE). Table 2 lists TRAEs that occurred in ≥10% of patients. The most common (≥25%) hematologic toxicities were leukopenia (83%), neutropenia (67%), thrombocytopenia (50%), and anemia (33%). Nonhematologic laboratory abnormalities included increased alkaline phosphatase (58%), increased alanine aminotransferase (ALT) and aspartate aminotransferase (AST; 58%), increased lipase (33%), increased creatinine (33%), and increased amylase (25%). Common nonlaboratory TRAEs included fatigue (58%), rash (58%), headache (42%), flu-like symptoms (33%), cough (25%), nausea (25%), pruritis (25%), and hepatitis (25%). Leukopenia and anemia were the only grade 3 toxicities that occurred in more than one patient (two patients each). Grade 3 neutropenia, hepatitis/elevated AST/ALT, flu-like symptoms, arachnoiditis, back pain, febrile neutropenia, and hypokalemia each occurred in one patient. Elevated lipase was the only grade 4 TRAE.

**Table 1.** Patient characteristics.

*n*	12
Age	​
Median	65.3
Range	44.2–92.9
Sex	​
Male	8
Female	4
Race/ethnicity	​
White non-Hispanic	11
Asian	1
Primary tumor site	​
Intraabdominal/retroperitoneal	11
Spermatic cord	1
Eastern Cooperative Oncology Group performance status	​
0	10
1	2
Number of prior systemic therapies	​
0	8
1	2
2	2

**Table 2.** TRAEs occurring in ≥10% of patients.

AE	Grade				
	1	2	3	4	Any
Leukopenia	5 (42)	3 (25)	2 (17)	0	10 (83)
Neutropenia	0	7 (58)	1 (8)	0	8 (67)
Fatigue	7 (58)	0	0	0	7 (58)
Alkaline phosphatase increased	6 (50)	1 (8)	0	0	7 (58)
Rash	4 (33)	3 (25)	0	0	7 (58)
ALT increased	3 (25)	3 (25)	1 (8)	0	7 (58)
AST increased	3 (25)	3 (25)	1 (8)	0	7 (58)
Thrombocytopenia	6 (50)	0	0	0	6 (50)
Headache	3 (25)	2 (17)	0	0	5 (42)
Creatinine increased	4 (33)	0	0	0	4 (33)
Flu-like symptoms	3 (25)	0	1 (8)	0	4 (33)
Lipase increased	2 (17)	1 (8)	0	1 (8)	4 (33)
Anemia	0	2 (17)	2 (17)	0	4 (33)
Cough	3 (25)	0	0	0	3 (25)
Nausea	3 (25)	0	0	0	3 (25)
Pruritus	3 (25)	0	0	0	3 (25)
Amylase increased	0	3 (25)	0	0	3 (25)
Hepatitis	0	2 (17)	1 (8)	0	3 (25)
Dysgeusia	2 (17)	0	0	0	2 (17)
Myalgia	2 (17)	0	0	0	2 (17)
Vomiting	2 (17)	0	0	0	2 (17)
Anorexia	1 (8)	1 (8)	0	0	2 (17)
Diarrhea	1 (8)	1 (8)	0	0	2 (17)
Lymphocyte count decreased	0	2 (17)	0	0	2 (17)

All counts represent *n* (%).

Four of 12 patients required dose delays of palbociclib due to neutropenia, all of which resolved with temporary dose delays culminating in count recovery. Five patients (42%) had clinically significant immune-related AEs (irAE) that were treated with corticosteroids. Three patients had hepatitis, one had grade 2 synovitis and a grade 2 rash, one had grade 2 nephritis and a grade 2 rash, and one had grade 3 arachnoiditis. All irAEs resolved with retifanlimab discontinuation and corticosteroid administration. Three of five patients who discontinued retifanlimab were continued on palbociclib alone after resolution of their toxicities. The patient with synovitis and a concurrent rash subsequently developed hepatitis on palbociclib monotherapy, which responded to corticosteroids. The median time to irAE occurrence was 35 days (range, 14–273). Due to the number and type of irAEs, further enrollment on the study was halted after a total of 12 patients were treated. The study was amended to change the dosing schedule, as emerging data found fewer toxicities with a concomitant rather than a sequential dosing strategy of CDK4/6i plus ICB therapy (28). The larger amended study will be reported separately.

Whereas formal analysis of the primary endpoint was not feasible in this cohort due to early termination of enrollment, the best responses by RECIST v1.1 were one patient with a PR, eight with stable disease (SD), and two who had progressive disease (PD); the other patient’s response was not evaluable (Fig. 1C and D). The median PFS and OS were 7.1 months [95% confidence interval (CI), 3.5–not reached (NR)] and 26.8 months (95% CI, 21.8–NR), respectively (Fig. 1E; Supplementary Fig. S1).

### scRNA-seq reveals a heterogenous tumor microenvironment and changes in cell type proportions with treatment

**Figure 2.**
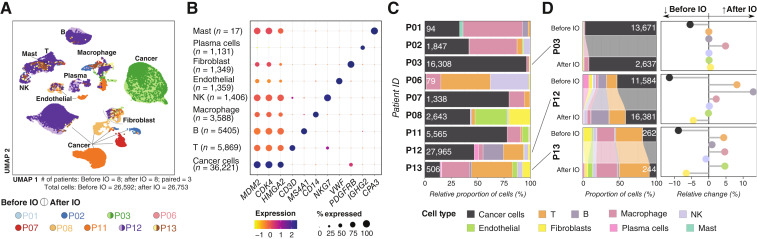
Overview of scRNA-seq data. **A,** Cell clustering by UMAP. **B,** Expression of key markers within major cell populations, pooled among all specimens. **C,** Cell type proportion in each patient. Patients with paired biopsies were merged. **D,** Comparison of cell type proportions before vs. after retifanlimab initiation (IO) in patients with paired biopsies. UMAP, Uniform Manifold Approximation and Projection.

Of the first 12 patients treated with sequential palbociclib and retifanlimab, nine had at least one tumor biopsy subjected to scRNA-seq (Fig. 2A). Nevertheless, three patients had paired biopsies available for analysis (P03, P12, and P13), two (P07 and P08) had biopsy samples pre-retifanlimab only, and four (P01, P02, P06, and P11) had post-retifanlimab biopsies only. Biopsies were not obtained at every time point for every patient due to concerns about patient safety, logistical constraints for the patient, or limitations in the laboratory capacity to process the samples. In total, transcriptomes from 56,345 cells were profiled, of which 36,221 tumor cells were identified based on joint overexpression of *MDM2*, *CDK4*, and *HMGA2*. The remaining 20,124 cells were classified using canonical gene expression markers, and represent immune cell subsets, endothelial cells, and fibroblasts (Fig. 2B). The proportion of each cell type within each core biopsy varied among patients; in six patients, >50% of cells were noncancer cells (Fig. 2C).

Cancer cells from each patient clustered independently from one another, highlighting the interpatient heterogeneity of such cells. Paired pre- and post-retifanlimab samples from the same patient clustered together. On the other hand, noncancer cells tended to cluster by cell type without discernible differences according to treatment or patient (Fig. 2A). Both cancer and noncancer cell proportions varied among patients and biopsy samples (Fig. 2C). Thus, blindly integrating samples as a cohort related to treatment may hinder identification of response and resistance mechanisms in highly genetically diverse tumor types. Furthermore, the number of cells obtained in each biopsy core varied, either because of inherent biological differences or limitations in sampling technique, further limiting comparative analyses between cores.

To identify changes in cell proportion across time, we examined the absolute change in the proportion of cancer and noncancer cell fractions in patients who had paired biopsy samples (Fig. 2D). All three patients had an absolute decrease in cancer cell proportion at the post-retifanlimab time point (range, −5.4% to −11.5% absolute decrease). P03 had no detectable T cells in either biopsy sample but had an increased macrophage fraction at the post-retifanlimab time point. In contrast, the paired P12 and P13 samples showed increases in T- (8.2% and 4.4%, respectively) and B-cell fractions (13.0% and 0.93%) but a decrease in fibroblast (−6.6% and −4.5%) fraction after initiating retifanlimab.

Additionally, we classified cancer cells based on their dominant gene expression signature, as defined by Gruel and colleagues (24). Most tumors had one or two dominant signatures accounting for more than 80% of cells (Supplementary Fig. S2A–S2C). Signature proportions shifted in response to treatment. For example, patient P03 (best response of PD) had an increase in cells with ECM remodeling and invasion signatures and a reduction of its dominant hypoxia signature after retifanlimab initiation (Supplementary Fig. S2D). Patient P12 (best response of SD) had decreased ECM remodeling signature after retifanlimab initiation and marginal changes in the invasion signature relative to the pre-retifanlimab time point. Changes in P13 were not significant, most likely due to a small number of cells.

### Longitudinal scRNA-seq analyses of paired biopsy samples identify features of resistance to palbociclib and retifanlimab

**Figure 3.**
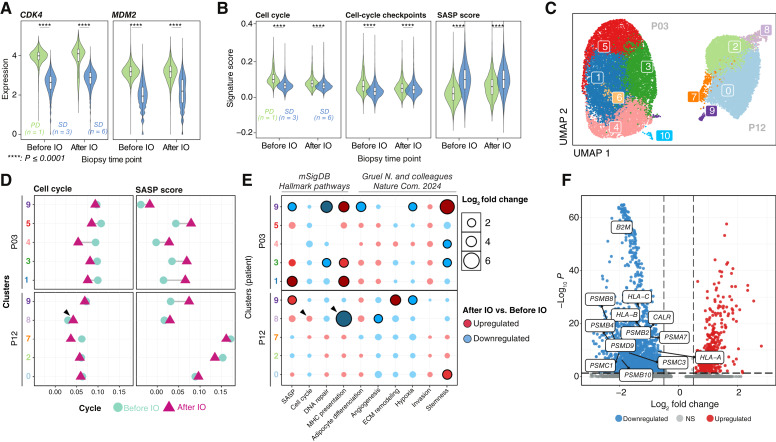
Changes in tumor and immune cell populations and phenotypes with anti–PD-1. **A,***CDK4* and *MDM2* expression in tumor cells according to best response. PD, P03, before and after retifanlimab initiation (IO); SD, three before IO and six after IO. **B,** Signature scores in tumor cells according to best response. Same samples as in **A**. **C,** Integrated clustering of cancer cells from patients with paired samples and sufficient tumor fraction. **D** and **E,** Intracluster signature score changes with initiation of retifanlimab, plotted (**D**) along the *x*-axis or (**E**) as dot size. **F,** Volcano plot of differential expression of genes related to immune response in cluster 8 vs. all other clusters from P12.

To determine whether radiographic response was associated with activity of tumor pathways targeted by therapy, such as cell cycle or immune modulation, we compared tumor samples from patients with a best response of SD to those with PD. The single patient with PR did not have a biopsy sample for analysis. P03, the only patient with a best response of PD, showed elevated *CDK4* and *MDM2* expression before and after retifanlimab initiation compared with patients with SD (Fig. 3A). P03 also had lower cell-cycle pathway transcripts, lower G1/S checkpoint activity, and lower SASP scores before retifanlimab initiation (Fig. 3B). Furthermore, P03 had no detectable lymphocytes or B cells before or after retifanlimab initiation. These findings may indicate that P03, who failed to achieve SD, had less palbociclib-induced cell-cycle arrest and a limited cell-mediated intratumoral inflammatory response at baseline and after PD-1 inhibition.

We next evaluated changes in cancer cell gene expression in paired samples from patients P03 and P12 (patient P13’s samples had insufficient cancer cells for further analysis). Cancer cells from the two patients clustered separately from one another (Fig. 3C). SASP expression scores were increased after retifanlimab initiation in nearly all clusters, whereas cell-cycle pathway scores decreased in all clusters except 1 (cluster 8; Fig. 3D and E). Cluster 8 also displayed decreases in antigen processing and presentation and adipogenesis signature scores after retifanlimab initiation (Fig. 3E; Supplementary Table S4). Differential gene expression analysis of cluster 8 versus other clusters from P12 revealed downregulation of key cell-cycle and immune-related gene transcripts, including *CDKN2A*, *B2M*, *HLA-A*, *HLA-B*, and *HLA-C*, whereas *MDM2*, *HMGA2*, and *CDK6* were upregulated (Fig. 3F; Supplementary Table S5). Thus, cluster 8 cells upregulated genes associated with the cell cycle and downregulated genes associated with antigen processing and presentation, representing possible resistance mechanisms to palbociclib and retifanlimab.

### Analysis of tumor-infiltrating and peripheral blood T-cell dynamics highlights changes in T-cell subsets after palbociclib and retifanlimab

**Figure 4.**
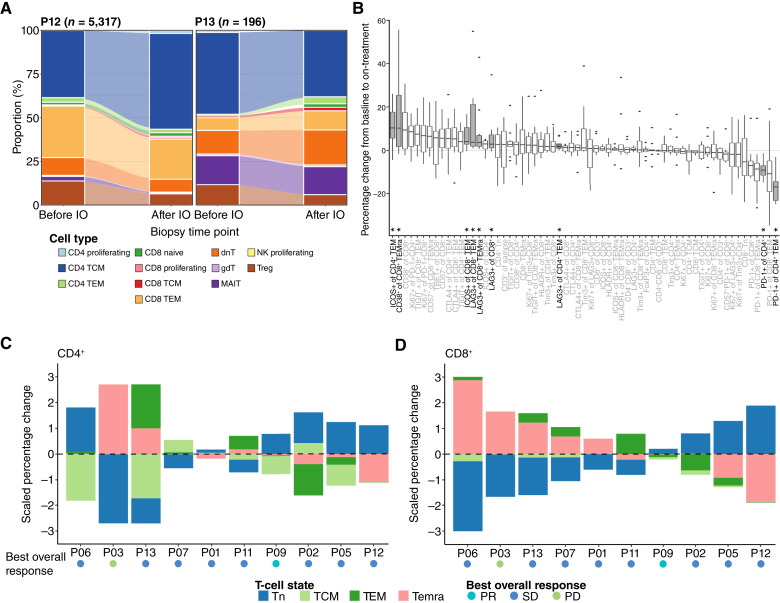
Changes in proportions of T-cell populations with anti–PD-1 via high-parameter flow cytometry. **A,** Change in abundance of T-cell subtypes with initiation of retifanlimab in patients with paired biopsies and detectable tumor-infiltrating T cells. **B,** Boxplot depicting percent change post- vs. pre-retifanlimab in the abundance of 63 T cell types. Filled boxes with bold labels and asterisks indicate T cell subsets with significant changes (*P* < 0.05; FDR < 0.05). **C** and **D,** Percent change in four key subsets of (**C**) CD4^+^ and (**D**) CD8^+^ T cells. OR, overall response.

Previous studies have shown that CDK4/6i can skew T cells toward a stem- or memory-like phenotype, potentially priming them for response to ICB (14, 15). To examine changes in T-cell differentiation states with study treatment, we analyzed the proportions of more than 20 T-cell subsets before and after retifanlimab initiation in the two patients with paired tumor biopsies and detectable tumor-infiltrating T cells (P12 and P13). Whereas the abundance of most T-cell subsets either did not change over time or changed differently between the two patients, Tregs decreased and Tn and Tcm CD8^+^ T cells slightly increased after retifanlimab initiation in both (Fig. 4A).

We next sought to detect changes in peripheral blood T-cell dynamics by analyzing PBMCs from 10 patients who had paired blood samples available for analysis (before palbociclib and after combination therapy) and comparing T-cell subpopulation percentages. We identified 9 of 63 subpopulations that significantly differed in proportion over time after correcting for multiple hypothesis testing (Fig. 4A). For example, the proportion of PD-1+CD4^+^ T cells decreased after treatment, whereas the expression of activation markers such as ICOS and CD38 increased on select T-cell subsets. Similarly, expression of the exhaustion marker LAG-3 increased on select CD4^+^ and CD8^+^ cell subsets, including on Tem cells and Tem cells reexpressing CD45RA (Temra).

To determine whether peripheral blood T-cell states shifted after combination therapy, we examined changes in four key states, namely Tn, Tcm, Tem, and Temra cells, within individual patients. Whereas increases in Temra cells often coincided with decreases in Tn cells and *vice versa*, changes in T-cell states varied between patients and between CD4^+^ and CD8^+^ T cells (Fig. 4B and C). Examples of individual shifts in T-cell states are presented in Supplementary Fig. S3.

## Discussion

This phase II study investigated the combination of palbociclib and retifanlimab in patients with advanced DDLPS. Palbociclib was administered alone in a 2-week lead-in, followed by concomitant treatment with retifanlimab. There were no safety signals seen in the initial safety run-in, but after a total of 12 patients were treated, a higher-than-expected number of immune-mediated toxicities requiring corticosteroid use were noted and further accrual was halted. Similar findings were seen in the PACT study, a phase Ia/Ib study of the PD-L1 inhibitor LY3300054 in combination with abemaciclib. Like our study, a 2-week lead-in of abemaciclib monotherapy preceded LY3300054 treatment. Three of the first four patients treated on the PACT study experienced grade ≥3 elevated transaminases, requiring treatment cessation. The study was subsequently amended to administer abemaciclib and LY3300054 concurrently without an abemaciclib lead-in. Twelve subsequent patients tolerated treatment with no hepatotoxicity, suggesting that the sequence of the study combination was important in determining toxicity (28). Others have also pointed out that novel CDK4/6i plus anti–PD-1 dosing schedules, such as a short course of CDK4/6is to “prime” the immune system prior to PD-1 inhibition, are worthy of further exploration (29). These observations, across both studies, reinforce the importance of rational sequencing and scheduling in future trials evaluating CDK4/6 and immune checkpoint inhibitor combinations not only in sarcoma but potentially other solid tumors as well.

Based on these results, our study (NCT04438824) was amended to administer palbociclib and retifanlimab concurrently on day 1; this study is ongoing, and the results will be reported separately. Because enrollment onto this dosing cohort was terminated early, the study was not adequately powered to evaluate efficacy. The median PFS of 7.1 months and the disease control rate of 75% are encouraging when compared with historic controls (8). However, the efficacy data must be interpreted with caution given the limited sample size.

To explore the biologic effects of CDK4/6 and PD-1 inhibition, we performed scRNA-seq on tumor biopsies collected during this study. Analysis of paired tumor samples revealed an increase in a DDLPS-specific gene signature associated with senescence in most cancer cells over time, highlighting the ability of CDK4/6i to push cells from quiescence into senescence (12). This approach also showed an increase in the proportion of intratumoral immune cells after retifanlimab administration, supporting our hypothesis of immune activation by the treatment combination. Furthermore, we identified a cluster of cancer cells in one patient that was seemingly refractory to combination therapy. This cluster was characterized by upregulation of cell-cycle genes and downregulation of antigen processing and presentation genes, suggesting inherent or acquired resistance to treatment. We next applied recently developed DDLPS-specific pathway analyses to cancer cells (24), highlighting dynamic changes in expression of dominant signatures such as hypoxia, ECM remodeling, and adipocyte differentiation.

In addition to tumor-based analyses, we performed flow cytometry on paired peripheral blood samples to identify pharmacodynamic changes. We identified an increase in Ki67+PD-1+ T cells after treatment, a finding previously described in patients with melanoma treated with ICB (30), an increase in activation and differentiation markers, and an increase in the exhaustion markers LAG-3 and TIM-3. The latter may represent compensatory inhibitor checkpoint expression after retifanlimab treatment. Additionally, CD57^+^CD8^+^ T cells increased after treatment, a subset previously described as senescent (31). Many of these findings were not statistically significant, possibly due to the small sample size in this dataset. Finally, whereas there were shifts in naïve and memory T-cell populations in both tumor and blood, the changes were not uniform, and investigation of these changes in a larger cohort may identify more meaningful trends. Given their minimally invasive and scalable nature, peripheral blood–based biomarkers could inform real-time immune monitoring strategies and early pharmacodynamic endpoints in future trials.

Our proof-of-concept work sets the stage for a larger analysis of tumor and blood samples collected on the amended version of this study, which administered palbociclib and retifanlimab concurrently (32). This study was limited by its lack of statistical power to assess clinical endpoints and a low number of paired tumor samples due to the early termination of enrollment at the initial dose and schedule. Some biopsies yielded a low number of cells, which may not precisely reflect the cell type diversity of the complete tumor. In addition, nonuniformity with respect to the timing of sample collections between the tissue and blood samples made comparisons of baseline tumor and blood samples challenging. Other groups have shown that expansion of memory T cells after initiation of CDK4/6i dissipates after introduction of PD-1 blockade (33) and that increases in Ki67+ PD-1+ T cells occur as early as 1 week after ICB but decline over time (34). Therefore, the timing of tissue collection is crucial in understanding the dynamics of T-cell subsets. Frequent blood sampling in future studies would be ideal to ascertain T-cell evolution after combination therapy.

In summary, our findings emphasize the need for optimized sequencing of CDK4/6 plus PD-1 inhibition, demonstrate the feasibility and utility of scRNA-seq on core biopsy samples from patients treated on clinical trials, and highlight the potential of peripheral blood immune monitoring to measure pharmacodynamic changes in response to treatment. An additional 30 patients have been enrolled to an amended version of this study under a revised dosing schema, with tumor and blood samples collected pre- and on-treatment. Analyses of this larger cohort will seek to validate these pharmacodynamic findings and determine the association of changes with clinical outcomes. This combination strategy may ultimately be applicable beyond liposarcoma as a means of inducing inflammation in traditionally “immune-cold” malignancies and enhancing responsiveness to immunotherapy.

## Supplementary Material

Supplementary Table S1 Supplementary Table S1. Flow cytometry antibodies.

Supplementary Table S2 Supplementary Table S2. Cohort clinical features.

Supplementary Table S3 Supplementary Table S3. Representativeness of Study Participants

Supplementary Table S4 Supplementary Table S4. Intra-cluster differential pathway analysis between pre- and post-retifanlimab biopsies

Supplementary Table S5 Supplementary Table S5. Transcriptome-wide differential gene expression between cluster 8 of P12 vs. all other P12 clusters.

Supplementary Figure S1 Supplementary Figure S1. Survival. A) Kaplan-Meier curve for progression-free survival. B) Kaplan-Meier curve for overall survival.

Supplementary Figure S2 Supplementary Figure S2. Classification of cancer cells according to WD/DD liposarcoma signatures.

Supplementary Figure S3 Supplementary Figure S3. T cell state contour plots.

## Data Availability

Raw and final gene count matrices are accessible via Gene Expression Omnibus accession # GSE320212.
