# Supplementary material for: Tumor and Immune Dynamics Following Sequential CDK4/6 and PD-1 Inhibition: Results from a Phase 2 Study in Dedifferentiated Liposarcoma
Source: Cancer Res Commun. 2026 Feb 27;6(2):437–46. doi: 10.1158/2767-9764.CRC-25-0334 (PMC13037771; doi:10.1158/2767-9764.CRC-25-0334)

Supplementary Figure S1. Survival. **A)** Kaplan-Meier curve for progression-free survival. **B)** Kaplan-Meier curve for overall survival.

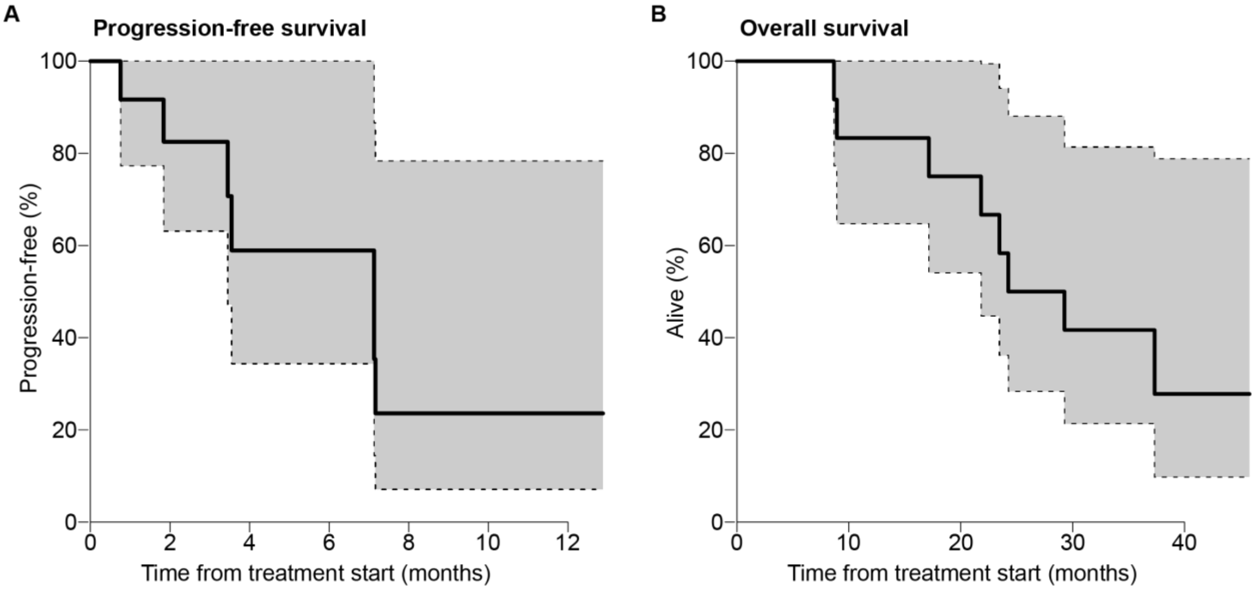

Supplement: Supplementary Figure S1 — Survival. A) Kaplan-Meier curve for progression-free survival. B) Kaplan-Meier curve for overall survival. [file crc-25-0334_supplementary_figure_s1_suppsf1.pdf]
