# Supplementary material for: Tumor and Immune Dynamics Following Sequential CDK4/6 and PD-1 Inhibition: Results from a Phase 2 Study in Dedifferentiated Liposarcoma
Source: Cancer Res Commun. 2026 Feb 27;6(2):437–46. doi: 10.1158/2767-9764.CRC-25-0334 (PMC13037771; doi:10.1158/2767-9764.CRC-25-0334)

Supplementary Figure S2. Classification of cancer cells according to WD/DD liposarcoma signatures. **A)** UMAP projection of cancer cells color coded by the dominant signature class. **B)** Bar plot depicting the proportion of cells expressing each signature in each sequenced biopsy. **C)** UMAP projections of cancer cells expressing each of the 6 WD/DD liposarcoma signatures separately. Cells with a signature score ≤ 0.4 re-colored in gray. **D)** Violin plots comparing expression of each signature between paired and unpaired pre- and post-retifanlimab biopsies.

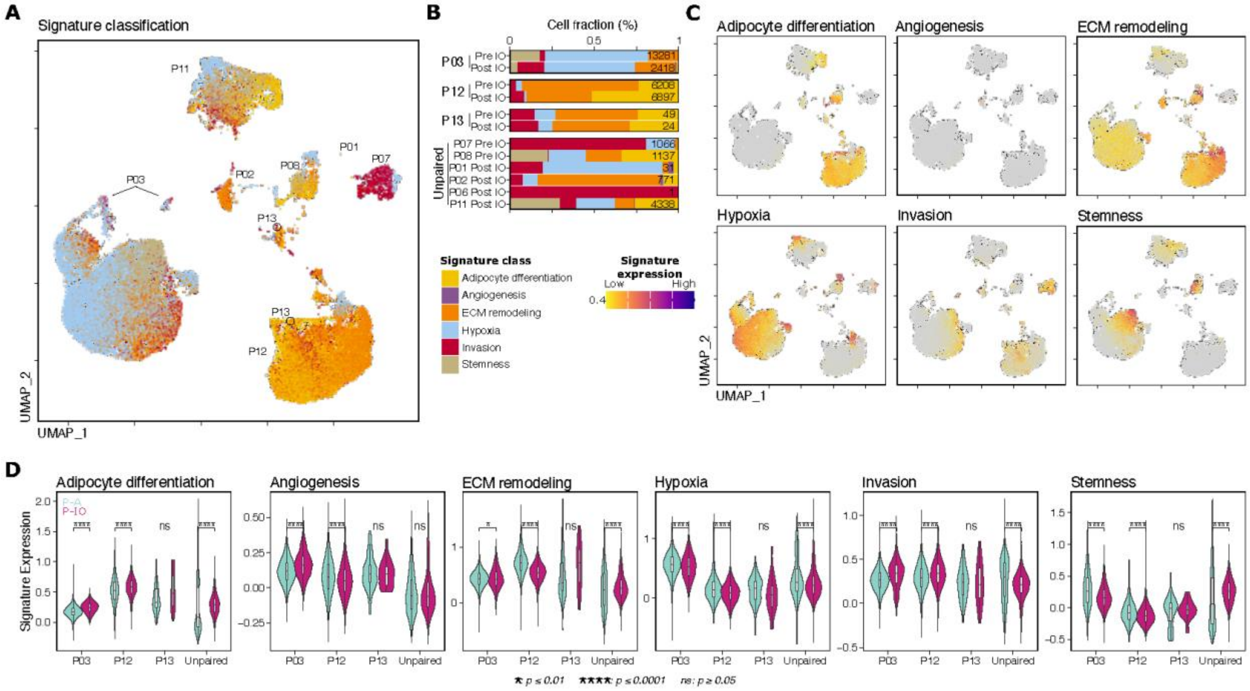

Supplement: Supplementary Figure S2 — Classification of cancer cells according to WD/DD liposarcoma signatures. [file crc-25-0334_supplementary_figure_s2_suppsf2.pdf]
