# Supplementary material for: Tumor and Immune Dynamics Following Sequential CDK4/6 and PD-1 Inhibition: Results from a Phase 2 Study in Dedifferentiated Liposarcoma
Source: Cancer Res Commun. 2026 Feb 27;6(2):437–46. doi: 10.1158/2767-9764.CRC-25-0334 (PMC13037771; doi:10.1158/2767-9764.CRC-25-0334)

Supplementary Figure S3. T cell state contour plots. Distribution of CD4+ and CD8+ T cells among 4 key states from **(A)** P06 and **(B)** P12 in pre- and post-retifanlimab peripheral blood. Dominant T cell state changes are indicated by pink arrows.

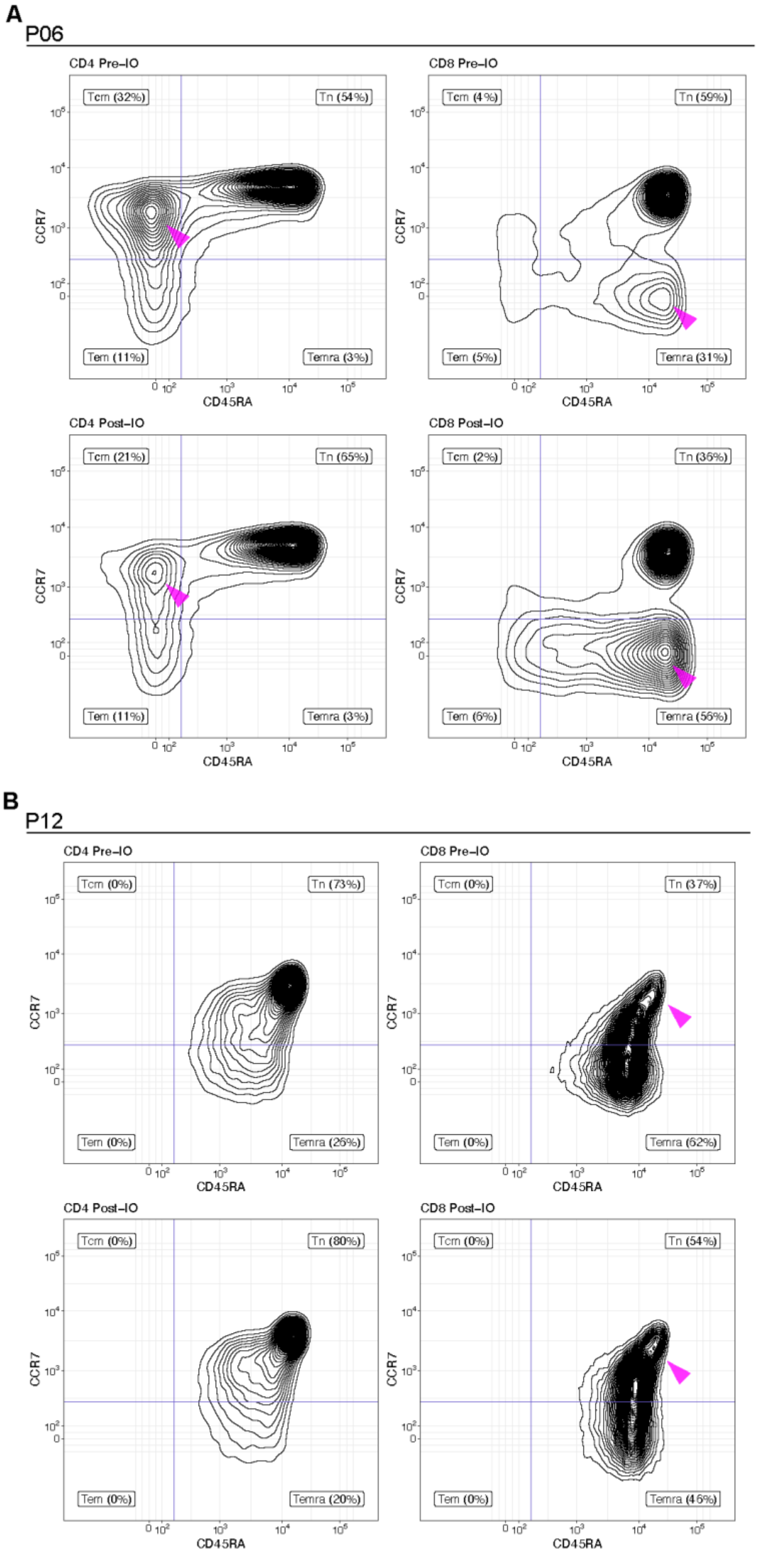

Supplement: Supplementary Figure S3 — T cell state contour plots. [file crc-25-0334_supplementary_figure_s3_suppsf3.pdf]
